# Drought as a trigger of the rapid rise of professional skateboarding in 1970s Southern California

**DOI:** 10.1093/pnasnexus/pgad395

**Published:** 2023-12-12

**Authors:** Ulf Büntgen, Clive Oppenheimer, Paco Li, Michael Frachetti, Jan Esper, Max C A Torbenson, Paul J Krusic

**Affiliations:** Department of Geography, University of Cambridge, Cambridge CB2 3EN, UK; Global Change Research Institute of the Czech Academy of Sciences, Brno 603 00, Czech Republic; Swiss Federal Research Institute (WSL), Birmensdorf 8903, Switzerland; Department of Geography, Faculty of Science, Masaryk University, Brno 613 00, Czech Republic; Department of Geography, University of Cambridge, Cambridge CB2 3EN, UK; Department of Geography, University of Cambridge, Cambridge CB2 3EN, UK; Department Anthropology, Washington University, St Louis, MO 63130-4899, USA; Global Change Research Institute of the Czech Academy of Sciences, Brno 603 00, Czech Republic; Department of Geography, Johannes Gutenberg University, Mainz 55099, Germany; Department of Geography, Johannes Gutenberg University, Mainz 55099, Germany; Department of Geography, University of Cambridge, Cambridge CB2 3EN, UK

**Keywords:** climate change, cultural history, drought extremes, environmental change, historical climatology, human behavior, interdisciplinary research

## Abstract

In 1977 California, authorities responded to an extreme drought with an unprecedented state order to drastically reduce domestic water usage and leave countless newly built swimming pools empty. These curved pools became “playgrounds” for inspired surfers to develop professional vertical skateboarding in the Los Angeles area. Industrial production of polyurethane, and the advent of digital photography, laser printing, and high gloss mass media further contributed to the explosive popularization of skateboarding, creating a global subculture and multibillion-dollar industry that still impacts music, fashion, and lifestyle worldwide. Our interdisciplinary investigation demonstrates that neither the timing nor the location of the origin of professional skateboarding was random. This modern case study highlights how environmental changes can affect human behavior, transform culture, and engender technical innovation in the Anthropocene.

## Introduction

Identifying societal and environmental factors that have influenced human history requires rational interpretation of diverse strands of reliable evidence. Cross-disciplinary studies drawing on combinations of instrumental and proxy-based climate data, as well as archaeological, historical, and econometric evidence, may range from assessments of societal “collapse” in deep history [1] (for which the depth of underpinning arguments is often limited) to considerations of environmental triggers of social unrest in modern times [2] (for which the importance of interacting factors is often unclear). Far less prevalent in scientific literature are investigations of the influence of climate change on popular culture and sport.

Here, we present a holistic perspective on the rise of professional skateboarding that examines why the sport and its attendant cultural and behavioral qualities developed in Southern California in the 1970s and not somewhere else before or after. Inspired by the 2001 documentary film *Dogtown and Z-boys* (directed by Stacy Peralta), our aim is to expose the complex spatiotemporal codependencies of a variety of environmental, technological, and societal factors that acted together to propel a local phenomenon into a global subculture and industry.

## The rise of skateboarding

In the 1950s, California became the worldwide center for a vibrant surf community, with local hotspots established along the Pacific coastline between San Diego in the south and San Francisco in the north. Malibu Beach and other places in the greater metropolitan area of Los Angeles (LA) were the most progressive foci where young surfers expanded into urban space and influenced music, fashion, and lifestyle. At the same time, the first printed color magazines offered a new form of mass media. Devoted to surfing and surf culture in Southern California, *The Surfer* magazine was first published in 1962 and became the community's “Bible,” inspired locally but exported globally.

Meanwhile, thanks to chemical engineers such as Otto Bayer, the industrial production of polyurethane became widespread in the 1950s, underpinning a revolution in sport articles (including skateboard wheels later). By the 1960s, increasing economic wealth, decreasing costs of living, and radical urban planning across much of the United States prompted an explosion in the installation of private and public outdoor swimming pools, with >150,000 pools constructed in California in the 1960s [3]. At its peak, up to 20,000 new kidney-shaped pools were installed per year in the greater LA region, accompanying a housing boom of single-family home construction. The “American Dream” was in full swing [4]. Soon, the new, curved-walled swimming pools in the suburbs of LA accounted for 60% of all pools in California [3, 5].

**Fig. 1.**
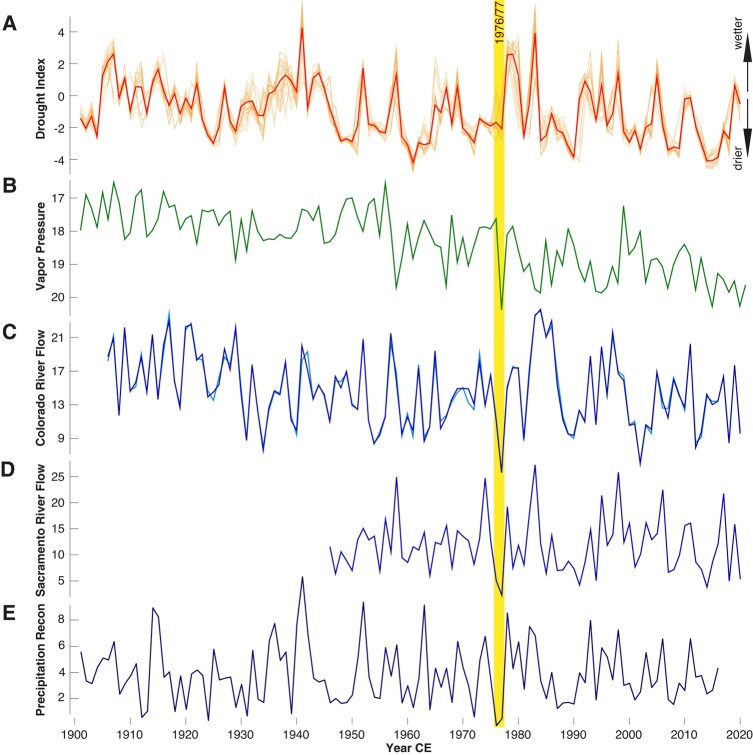
Hydroclimatic changes in the western United States over the 20th and early 21st centuries. A) Drought variability from 1901 to 2020 based on the self-calibrated Palmer Drought Severity Index and averaged over a smaller and larger region (33–34°/32–35° N and 117–119° W), with monthly and annual means shown in pale and intense, respectively. B) August atmospheric vapor pressure from 1901 to 2021 averaged over 32–35° N and 117–119° W (note reversed scale). C) Naturalized Colorado River flow measured at Lees Ferry, Arizona (36°52′03″ N and 111°35′43″ W), calculated for the water (before October to current September) and calendar years from 1906 to 2021, and expressed in thousands of acre-feet. D) Naturalized Sacramento River flow measured at Delta, California (40°56′23″ E and 122°24′58″ W), calculated for the water year (before October to current September) from 1946 to 2020, and expressed in thousands of acre-feet. E) Tree ring-based reconstruction of heavy (storm-delivered) precipitation totals (mm × 100) in Northern California [6]. Data availability as follows: A and B) at https://climexp.knmi.nl/; C) at https://www.usbr.gov/; D) at https://waterdata.usgs.gov/; and E) at https://www.ncdc.noaa.gov/.

Following these technical and infrastructural developments, California experienced a decade of prolonged aridity (Fig. 1A), with 1976/1977 being the driest consecutive 2 years of the 20th century (Fig. 1B–E). The extreme water scarcity in 1977 was amplified by unusually high summer temperatures that increased the atmospheric vapor pressure and exacerbated the hydrological deficit of previous years. Reflecting this rare coincidence of high temperature and low precipitation, the 1976/1977 drought resulted in exceptionally low flow rates of the Colorado and Sacramento Rivers which represent California's most important internal and external water sources (Fig. 1C and D), respectively. While this drought is unexceptional in a millennial context [7] [and California is historically known as a drought-prone state that experienced severe “Water Wars” in the past [8]], 1976 was nevertheless the fourth driest, and 1977 the driest winter-spring season ever recorded in northern California [9]. Moreover, 1976/1977 were the most extreme consecutive years of low December–April precipitation reconstructed over the past 440 years [6], coinciding with an extraordinary lack of winter storms [10]. California's climate is among the most variable of any United States state, and 1977 was its driest year by then.

The prolonged 1976/1977 drought resulted in estimated losses of 3 billion US Dollars in the state's agricultural sector [11, 12]. California's reservoir storage reached a record low in 1977 and water agencies mandated severe cuts in allotments [12]. The state government proposed a series of laws to increase the efficiency and flexibility of governance at lower political levels to accelerate the response to climate crises. The Metropolitan Water District of Southern California responded by transferring >320,000 acre-feet of water (nearly 400 million m^3^) to the more drought-affected central and northern parts of the state [11, 12]. Widespread water conservation measures included the prohibition of the filling of swimming pools [11]. The combination of innovative suburban planning and drastic drought legislation left more pools empty in LA than in any other part of the United States [3].

**Fig. 2.**
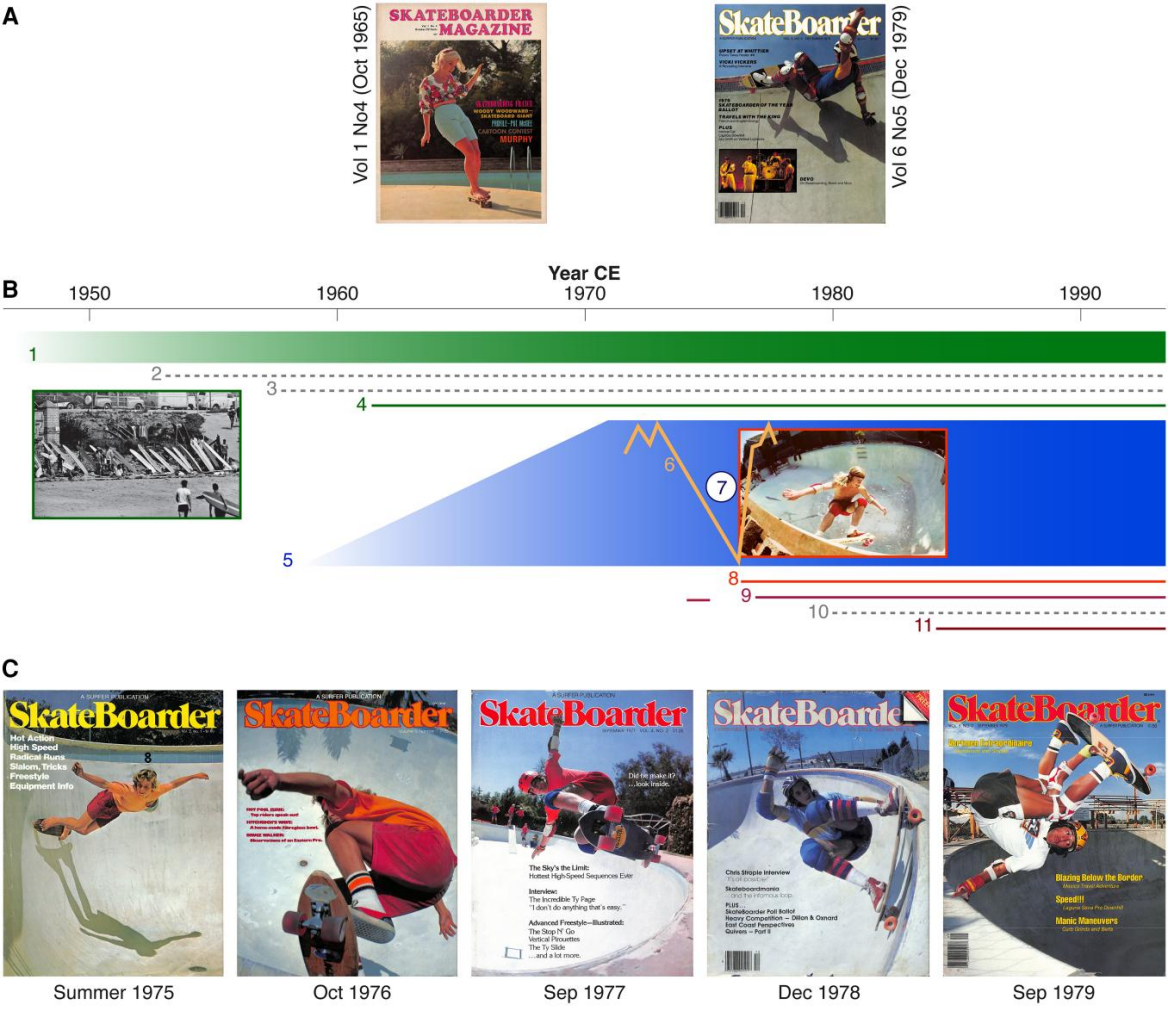
Interplay of environmental and social factors at the rise of professional skateboarding. A) First covers of the *Skateboarder* journal from October 1965 when pools were still filled with water, and skateboarding was more an amusement than a sport. B) Surf culture in Southern California (1), printed color magazines as mass media (2), production of polyurethane (3), publication of *The Surfer* magazine (4), development of tens of thousands of new pools in the greater LA region (5), mega drought across the Western United States (6), empty pools in Southern California (7), start of vertical pool-skating (8), foundation and establishment of major skateboard companies (9), invention and commercial production of digital cameras and video recorders (10), and beginning of first commercial skateboard videography (11). C) Cover images of the early *Skateboarder* magazine show the technical and acrobatic evolution of skateboarding from 1975 to 1979.

The confluence of a vibrant surf community and an initial freestyle skateboard scene including early pioneers such as Rodney Mullen, together with empty, kidney-shaped swimming pools, urethane-based skateboard wheels with high-performance ball bearings, as well as thriving entrepreneurship, formed a unique socio-environmental backdrop to the rapid rise of professional skateboarding in the mid-1970s in Southern California (Fig. 2). Following the advent of amateur skateboarding in the 1960s (Fig. 2A), the local surf and rising skate communities discovered new “playgrounds and materials” to develop vertical pool-skating within a few years [13, 14]. Originating as a teenage hobby in the 1960s (see *The Devil's Toy*; one of the first skateboard films produced in 1966), the early dawn of freestyle skateboarding evolved into professional halfpipe skateboarding through the formation of team sponsorships, the most famous of which was the *Zephyr* team (*Z-Boys*) in the “Dogtown” area of Santa Monica near Venice Beach.

The early cover images of the *Skateboarder* magazine between 1975 and 1979 reveal the extraordinary pace of technical and acrobatic innovation during this period (Fig. 2C). While the summer cover of 1975 already shows an empty, round-shaped pool, skateboarding is still rudimentary, and the young Greg Waver is navigating barefoot on a slim and flat freestyle board without nose and tail. Dominated by freestyle and streetstyle skateboarding, it is the first issue that refers explicitly to vertical pool riding (i.e. “Getting Vertical”) and contains an advertisement for the *Zephyr* skateboarding team (i.e. “Getting Radical”). A year later, the sport had developed substantially—the October 1976 cover shows Tony Alva skating “above” the Soul Bowl pool in San Diego, and the issue offers unique insights into the commercialization and professionalization of the sport. Boards, wheels, trucks, and bearings have advanced considerably, and the extensive journal issue even dedicated a section to vertical skateboarding.

The September 1977 cover and issue reveal that not only skateboards but also gear and tricks have reached a new dimension. With Paul Hackett skating a pool in Malibu, and many examples of vertical skating, the journal issue demonstrates how professional skateboarding became increasingly gravity defying. The December 1978 cover shows Steve Olson in a pool at the Del Mar Skate Ranch, and the issue contains several examples of further developments in professional skateboarding. The September 1979 cover shows the fully equipped professional skateboarder Eddie Elguera riding “in and beyond” an empty pool. The issue provides much evidence of the popularity of vertical skateboarding at the expense of freestyle and streetstyle, and how a local trend sport has become a global industry. Compare this with one of the first covers of the *Skateboarder* journal from October 1965 when pools were still filled with water (Fig. 2A), and skateboarding was more an amusement than a sport! A closer look at the *Skateboarder* cover from December 1979 not only displays the continuing professionalization and marketing of skateboarding but also reveals its international popularity and the beginnings of its influence on the music scene.

Major skateboard companies were founded in this period, including *Vision Street Wear* in 1976, *Alva* in 1977, and *Powell Peralta* in 1978. The local hype of pool and street skateboarding between San Diego and LA was further amplified by the invention and production of digital cameras and consumer video cameras in the early 1980s, which enabled commercial skateboard videography. Early examples of true skateboard films (not films about or with skateboarding) include *Bones Brigade* (1984) and *Shackle Me Not* (1988). Comparisons with *The Devil's Toy* (1966) or *Skateboard: The Movie that Defies Gravity* (1978) reinforce the rapid acceleration of skateboarding in the late 1970s and 1980s. This was the sport's “Golden Age” when the first professional skaters became global media stars. Protagonists like Tony Hawk, Danny Way, and Tony Magnusson enhanced the sport and industry of skateboarding in the early 1980s through the creation of new tricks and styles, as well as the foundation of their own companies. Professional halfpipe skateboarding quickly arrived in Europe where the *Münster Monster Mastership* in Germany in the 1980s heralded international skateboard contests with thousands of spectators [15]. Known as the first World Cup in 1989, the event also became the first official World Championship in freestyle, streetstyle, and halfpipe skateboarding in 1990. Five years later, the *X-Games* extended the platform for professional skateboarding, which had its Olympic debut in 2021 in Tokyo, Japan, and continues to influence a global subculture and multibillion-dollar industry, including hip-hop music and streetwear fashion. The impact of skateboarding on snowboarding cannot be over-emphasized.

## Concluding remarks

Our investigation into the entanglements between cultural, commercial, environmental, and political factors in 1970s Southern California shows that neither the timing nor the geographic origin of professional vertical skateboarding was random (Fig. 2). Any drought before the 1970s could not have had the same effect, because (i) the many thousands of kidney-shaped pools with curved bottoms and walls were only built in the 1960s, (ii) polyurethane and ball bearings had only recently become commercially available, and (iii) digital photography, video cameras, laser printing, and colorful mass media did not exist. Moreover, California was the only place worldwide where an intertwined surf community and entrepreneurship were able to transform local freestyle skateboarding into professional halfpipe skateboarding. In analyzing several climate records and describing the causal linkages between a range of environmental and societal factors at the rise of professional skateboarding in the Southern California in the 1970s, our interdisciplinary study goes far beyond the public perception that this global sport and subculture simply arose during a mega drought.

Our findings demonstrate that even small environmental changes in the Anthropocene can have a profound influence on human behavior, and stimulate cultural and technical innovation if interacting factors coincide geographically and temporally. There have been many studies arguing for the role of climate change in understanding human history of the distant past, for which the resolution, quality, and/or dating of evidence is usually limited. The case we have examined here demonstrates the analytical strength of modern, and somehow unusual examples of the human–climate nexus, and we hope it will stimulate a greater interest in interdisciplinary studies of 20th and 21st century history and innovation, for which high-resolution environmental and societal observations abound.

## Data Availability

All data used in this study are freely available (see caption of Fig. 1).
